# Lamellar keratoplasty using acellular porcine corneal stroma for the treatment of corneal ulcers

**DOI:** 10.3389/fmed.2025.1534210

**Published:** 2025-03-17

**Authors:** Yiming Hu, Lan Zhang, Lei Wu, Yao Zhu, Li Wu, Chenye Li, Youyang Ruan, Yunwei Hu, Feifei Wang, Zhirong Lin, Qifang Jin

**Affiliations:** ^1^Ophthalmic Center, The Second Affiliated Hospital, Jiangxi Medical College, Nanchang University, Nanchang, China; ^2^Department of Ophthalmology, Tongcheng County People’s Hospital, Xianning, China; ^3^Department of Ophthalmology, Xichang People’s Hospital, Yichang, China; ^4^School of Optometry, Jiangxi Medical College, Nanchang University, Nanchang, China; ^5^The Affiliated Xiamen Eye Center of Xiamen University, School of Medicine, Xiamen University Xiamen, Xiamen, China

**Keywords:** acellular porcine corneal stroma, laminar keratoplasty, fungal corneal ulcer, viral corneal ulcer, corneal graft

## Abstract

**Objective:**

The study aimed to investigate the efficacy and safety of acellular porcine corneal stroma (APCS) for lamellar keratoplasty in the treatment of corneal ulcers.

**Methods:**

A total of 14 patients (14 eyes) diagnosed with corneal ulcers who underwent lamellar keratoplasty using acellular porcine corneal stroma at the Second Affiliated Hospital of Nanchang University between June 2016 and May 2017 were recruited and followed up for at least 12 months. Postoperative visual acuity, epithelial recovery, graft transparency, the recurrence rate of corneal ulcers, the rate of graft rejection, corneal neovascularization, graft infection, secondary glaucoma, and graft melting were examined and analyzed.

**Results:**

All 14 patients (100%) who underwent lamellar keratoplasty using acellular porcine corneal stroma successfully preserved the structure of their eyeballs. The visual acuity improved in 11 patients (78.5%). Graft rejection occurred in one patient (7.1%), while two patients (14.3%) developed recurrent corneal ulcers. Corneal vessel ingrowth was observed in seven patients (50%), and one patient (7.1%) developed pseudopterygium. The average time for complete epithelial recovery was 3–7 days.

**Conclusion:**

Lamellar keratoplasty using acellular porcine corneal stroma is an effective surgical alternative for the treatment of corneal ulcers.

## 1 Introduction

Corneal diseases are the second most common eye condition after cataracts, with a high risk of complications. Infectious corneal ulcers are a primary cause of corneal inflammation (1–3). Corneal blindness can be treated through corneal transplantation, which is highly dependent on the availability of corneal donor tissues (4). Due to the shortage of donor corneas in China (5), less than 10,000 patients are able to regain their vision through corneal transplantation each year, and only1 in 70 people has the opportunity to receive corneal transplantation (6–7). Many patients miss the optimal window for treatment while waiting for donor corneas. Therefore, increasing the availability of corneal donors is crucial to addressing corneal blindness in China. To address this issue, many researchers have explored the use of biomaterials, natural polymer materials, and synthetic polymers to synthesize artificial corneas. Among the various bioengineered options, porcine corneas have been extensively studied due to their similarity to human corneas in size, thickness, microstructure, and refractive ability (8). Numerous studies have confirmed that the decellularized porcine corneal matrix exhibits good histocompatibility and low immunogenicity (9–11). *In vitro* experiments, animal experiments, and clinical use have demonstrated their safety and effectiveness in treating corneal ulcers, offering a potential solution to the shortage of donor corneas (12–15) and addressing the issue of limited corneal donor availability in certain regions (16). In 2015, the world’s first commercial tissue-engineered cornea, acellular porcine corneal stroma (APCS), was approved by the China Food and Drug Administration (CFDA) as a clinical alternative to human corneas in lamellar corneal transplantation (17). APCS is prepared through a series of strictly controlled procedures to remove antigens such as cells, heteroproteins, and polysaccharides from the cornea while retaining the natural corneal matrix stromal structure. It serves multiple functions, including physical coverage, lubrication, isolation, and wound protection after being applied to the affected area. In addition, it can promote corneal epithelial regeneration and matrix synthesis and replace the superficial corneal tissue of the original lesion for lamellar corneal transplantation (18). Studies have shown that regardless of whether the corneal ulcer is located in the peripheral or central cornea, patients who underwent corneal transplantation using APCS as the graft experienced significant visual improvement at both 3 months and 6 months postoperatively (19). From June 2016 to May 2017, 14 patients (14 eyes) who underwent lamellar keratoplasty using APCS at our hospital were clinically analyzed to investigate the efficacy and safety of APCS.

## 2 Participants and methods

### 2.1 Participants

A total of 14 patients (nine males and five females) with an average age of 51.7 ± 4.9 years, all diagnosed with corneal ulcers, underwent lamellar keratoplasty using acellular porcine corneal stroma at our hospital between June 2016 and May 2017. Among these patients, eight had fungal keratitis (57.1%), four had viral corneal infections (28.6%), and two had bacterial infections (14.3%). The follow-up period ranged from 3 to 12 months. The study and surgical protocol were approved by the Ethics Committee of the Second Affiliated Hospital of Nanchang University and were conducted in accordance with the principles of the Declaration of Helsinki.

### 2.2 Criteria

#### 2.2.1 Inclusion criteria: the criteria included the following

Patients who were diagnosed with infectious corneal ulcers based on medical history, physical examination, and auxiliary tests; aged between 40 and 60 years old; and whose preoperative anterior segment optical coherence tomography (AS-OCT) combined with ultrasound biomicroscopy showed that the corneal lesions did not involve Descemet’s membrane. Slit-lamp examination showed non-significant hypopyon in the anterior chamber, and the diameter of the corneal ulcers ranged from 3 to 8 mm. After 1 week of anti-infective treatment, the lesion was not controlled and tended to expand. Ocular ultrasonography showed no obvious abnormal echo signals in the vitreous cavity of the infected eye. All patients or their families who fully understood the purpose and significance of this study and signed the informed consent form were included.

#### 2.2.2 Exclusion criteria: the exclusion criteria included the following

Full-thickness corneal infection or corneal perforation; corneal lesions located within 2 mm of the limbus; diameter of corneal lesions >8 mm; cornea neovascularization; neuroplastic corneal ulcer; decreased or loss of corneal sensation; a history of ocular surface diseases such as moderate to severe dry eye syndrome, palpebral fissure insufficiency, or systematic immune disorders; severe allergies; patients who were in poor physical condition and unable to tolerate surgery; mentally ill and religiously restrictive individuals; and those who were unable or unwilling to receive acellular porcine corneas. All surgeries were performed after obtaining written informed consent from the patients.

### 2.3 Preoperative medication

A total of eight cases of fungal corneal ulcers were treated with a topical 5% natamycin ophthalmic solution (Note: LiJing; North China Pharmaceutical Co., Ltd., China) at a frequency of once per hour and oral itraconazole capsules (Sporanox; Xian Janssen Pharmaceutical Co., Ltd., China) at a dose of 0.2 g once daily. In addition, one case of *Pseudomonas aeruginosa* corneal ulcer and one case of *Staphylococcus* corneal ulcer were treated with topical levofloxacin eye drops (Cravit; Santen, Japan) at a frequency of once per hour, tobramycin eye drops (TOBREX; Alcon Inc., Switzerland) once per hour, and systemic intravenous antibiotics. Furthermore, four cases of viral corneal ulcers were treated with topical ganciclovir eye drops (6 times/d) (Jingming; HuBei Yuanda TianTianming Pharmaceutical Co., Ltd., China) and intravenous acyclovir (Hidragon Pharma, China) for 1 to 3 weeks.

### 2.4 Surgical procedures

All surgeries were performed by the same experienced clinician (Dr. Qifang Jin) at our hospital. According to the diameters of the ulcer lesions, trephines with different diameters were selected to completely remove the diseased corneas. Then, the corneal stromal surface was irrigated with a 0.02% fluconazole solution or tobramycin solution, depending on the type of infection. The excised corneas were sent for pathological examination and pathogen culture for fungi and bacteria. Acellular porcine corneal stroma (Aixin Pupil, China Regenerative Medicine International Limited, China), cut with a trephine 0.5 mm larger in diameter than the recipient, was placed in normal saline for 1 min to rehydrate. The rehydrated decellularized porcine corneal matrix was then sutured intermittently to the graft bed with 10–0 nylon thread (non-absorbable surgical suture; Alcon Inc., United States) using 16–18 stitches.

### 2.5 Postoperative medication

0.3% sodium hyaluronate eye drops (WanHanRunMing; Wan Han Pharmaceutical Co., Ltd., China) (4 times/d) were used to promote epithelial recovery in all patients. Patients with fungal corneal ulcers received topical 5% nataloxacin eye drops (6 times/d), levofloxacin eye drops (6 times/d), pranoprofen eye drops (Qian Shou Pharmaceutical Co., Ltd., Japan) (4 times/d), and oral itraconazole capsules (0.2 g, once daily). Regular follow-up examinations were conducted. Once we confirmed that the patient had recovered well and there were no signs of infection, we began using tobramycin dexamethasone eye drops (QiLu Pharmaceutical Co., Ltd., China) (4 times/d), starting 1-month post-surgery to enhance the anti-inflammatory effect. Two months post-surgery, the frequency of the medication was gradually reduced from four times a day to three times a day, then to twice a day after 3 months, and to once a day after 6 months, continuing for more than 1 year. For the bacterial corneal ulcer, levofloxacin eye drops (6times/d), tobramycin dexamethasone eye drops (6 times/d), and intravenous antibiotics were continued, with the dose tapered when there was no recurrence. For the viral corneal ulcer, ganciclovir eye drops (6times/d), levofloxacin eye drops (6times/d), and tobramycin dexamethasone eye drops (4times/d) were continued. Intravenous acyclovir was continued and then changed to oral acyclovir (QiangNi; ShangDong QiDu Pharmaceutical Co., Ltd., China) (0.4 g twice daily) for 1–3 months.

### 2.6 Postoperative follow-up

Slit-lamp examination, best-corrected visual acuity (BCVA), intraocular pressure, and corneal fluorescein sodium staining were performed on day 1 and 1 week, 1 month, 3 months, 6 months, and 12 months after the surgery. Corneal transparency was scored and graded, and the recurrence rate of the primary infection, graft rejection rate, and corneal neovascularization were analyzed.

The criteria for the recurrence of corneal infection were as follows: aggravated symptoms of ocular irritation, such as pain and photophobia, reappearance of corneal ulcers in grafts, and aggravated edema of the corneal graft bed. Corneal transparency, corneal edema degree, and corneal neovascularization were observed under the slit-lamp microscope according to the Holland scoring criteria (20). Graft transparency was scored as follows: 0, the graft was transparent, without any turbidity or cloudiness; 1, mild opacity, the details of the iris were clearly visible; 2, moderate corneal opacity with visible pupil and iris through the cornea, but the details of the iris were not clear; 3, severe corneal opacity with invisible pupil and iris; and 4, corneal opacity, anterior chamber not visible. Graft edema was scored as follows: 0 point, no edema; 1 point, mild epithelial edema; 2 points, mild edema of both the corneal epithelium and stroma; 3 points, obviously edema of both the corneal epithelium and stroma; and 4 points, bullous keratopathy. The corneal neovascularization was scored as follows: 0 point, no neovascularization; 1 point, corneal graft bed neovascularization; 2 points, the corneal graft entry was less than 2 mm or the corneal limbus was less than 1 quadrant; 3 points, the corneal graft entry was more than 2 mm or the corneal limbus was more than 1 quadrant; and 4 points, neovascularization covered the cornea. The ocular irritation symptom scores were as follows: 0 points, no ocular irritation; 1 point, mild and tolerable eye irritation symptoms; 2 points, obvious but tolerable irritation symptoms affecting usual life or work; and 3 points, severe and intolerable irritation symptoms affecting daily life.

### 2.7 Statistical analysis

The data were imported into the Statistical Package for the Social Sciences (SPSS Inc., Chicago, IL, version 26.0) for analysis. Measurement data were presented as the mean and standard deviation. A paired *t*-test was used for comparison. A significance level of a *p*-value <0.05 was set.

## 3 Results

### 3.1 Postoperative recovery of the patients

**Figure 1:**
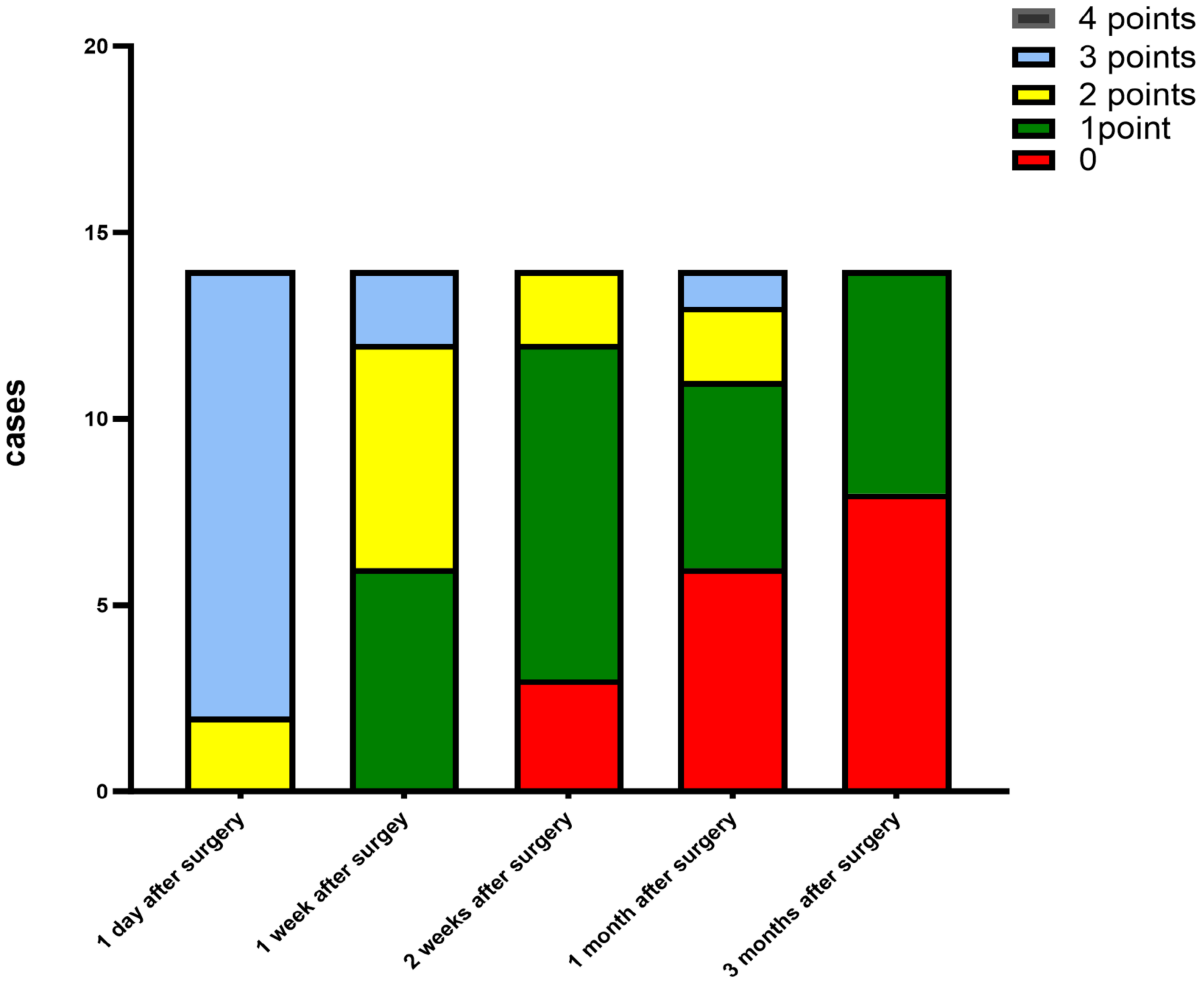
Distribution of the graft edema scores at each follow-up time point after the surgery.

**Figure 2:**
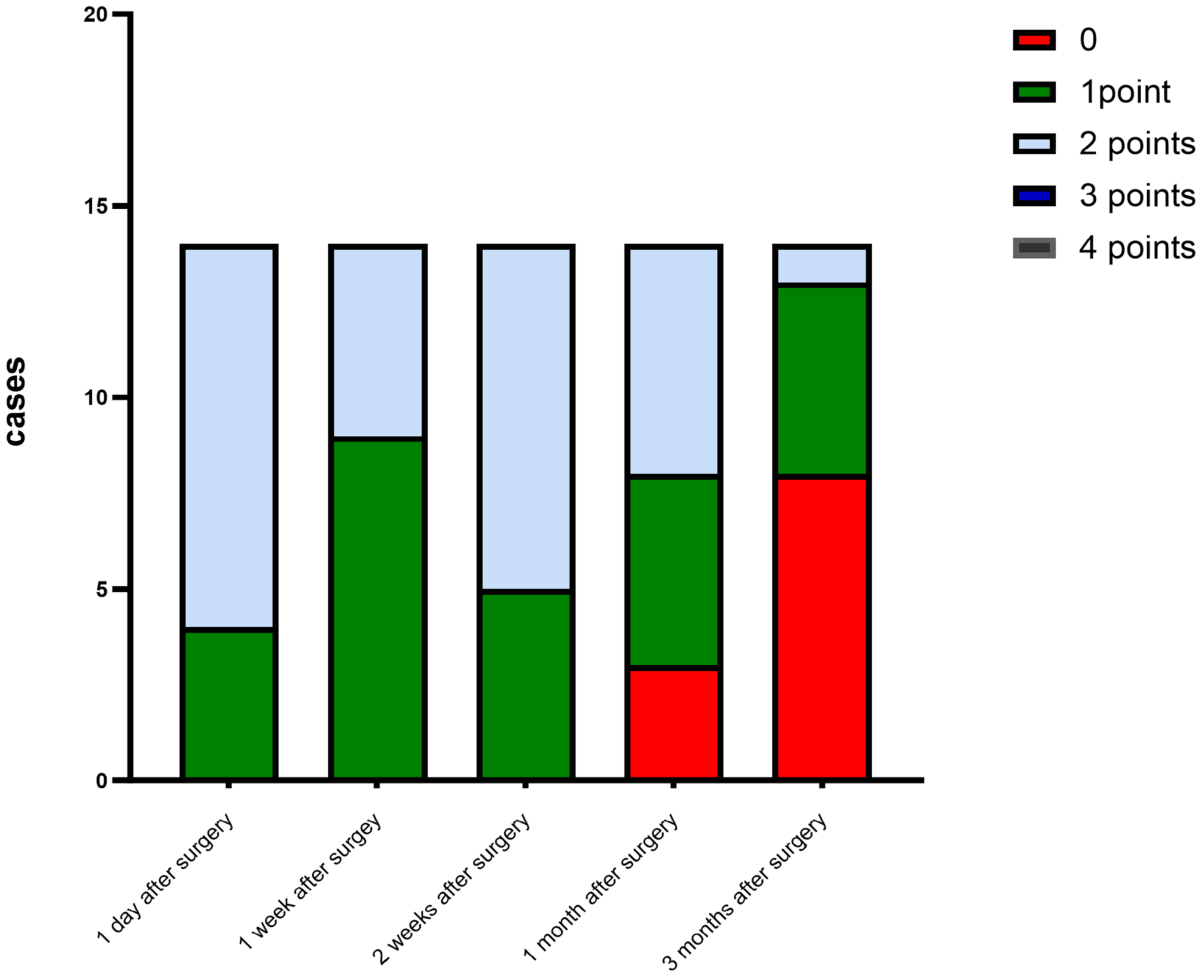
Distribution of the corneal transparency scores at each postoperative time point.

**Figure 3:**
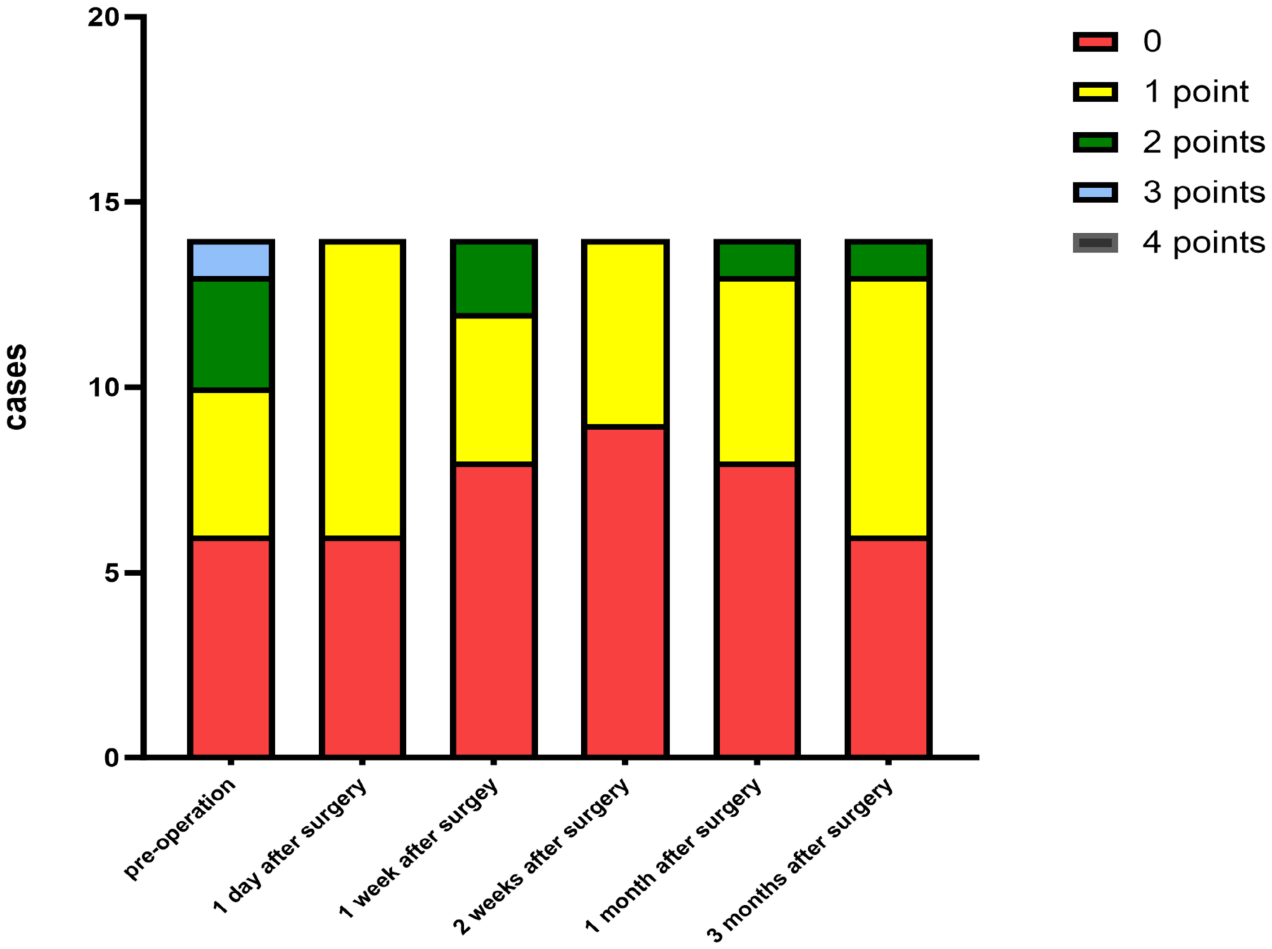
Distribution of the corneal neovascularization scores at each preoperative and postoperative follow-up time.

**Figure 4:**
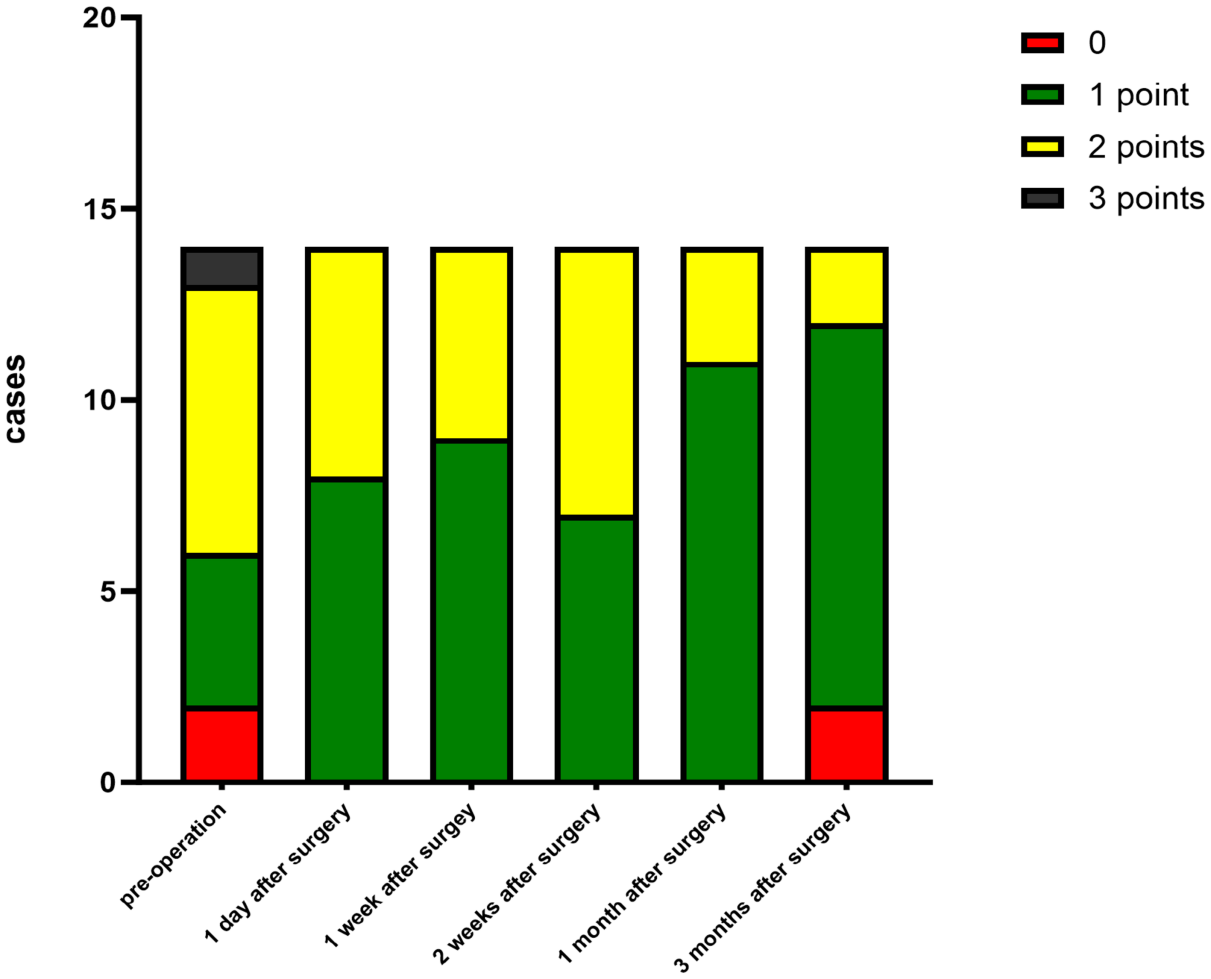
Distribution of the ocular stimulation scores at each preoperative and postoperative follow-up time point.

The corneal infections were controlled in all patients without corneal perforation, and the irritating symptoms were relieved after the surgery. The visual acuity improved in 11 patients (78.5%) at 3 months after the surgery (Table 1). The best-corrected visual acuity (BCVA) at 3 months after the surgery showed significant improvement compared to the preoperative values (t = 4.857, *p*<0.05). The corneal epithelium was completely healed within 3–7 (4.643 ± 1.151) days after the surgery. Under the slit-lamp examination, corneal edema generally decreased within 3 months after the surgery (Figure 1). Corneal edema and opacity were observed during the early postoperative period, and the corneas gradually restored their transparency within the following month (Figure 2). Corneal new vessels developed during the early postoperative period but regressed with the use of topical anti-inflammatory therapy (Figure 3). The ocular irritation symptoms in the patients gradually decreased after the surgery (Figure 4).

**Table 1:** Comparison of the patients’ visual acuity before and after the surgery at 3 months.

Serial number	Preoperative visual acuity	Visual acuity at 3 months postoperatively
1	HM	0.1
2	CF 10 cm	0.3
3	0.1	0.4
4	HM	0.2
5	0.1	0.1
6	0.02	0.08
7	0.2	0.2
8	0.2	0.4
9	0.06	0.3
10	CF 10 cm	0.08
11	0.1	0.1
12	HM	0.1
13	0.1	0.3
14	0.06	0.2

Decimal visual acuity for counting fingers (CFs) is quantified (VAS) as 0.0025, and the hand movement (HM) is equal to 0.002.

### 3.2 Postoperative complications

**Figure 5:**
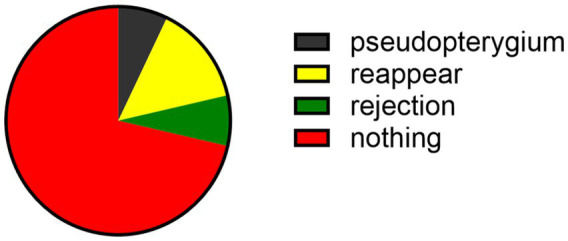
Postoperative complications.

A total of one patient (7.1%) had corneal graft rejection after the surgery, manifested as eye discomfort, red eyes, photophobia, and decreased vision. Under the slit-lamp examination, the conjunctiva was congested, the cornea was edematous and cloudy, and its transparency was reduced. Neovascularization grew at the suture site. Following conjunctival injection of 0.025% dexamethasone (0.3 milliliters), topical use of 0.1% tobramycin dexamethasone eye drops (every hour), and systemic intravenous injection of dexamethasone (10 milligrams daily), the rejection reaction was controlled and the transparency gradually recovered. The graft gradually restored the transparency. A total of two patients (21.4%) with viral infection had recurrence of corneal ulcers, which were controlled after antiviral therapy, and one patient (7.1%) developed pseudopterygium (Figure 5).

### 3.3 Typical cases

A1–A5 show images captured from a patient with a bacterial corneal ulcer in the right eye. The pathogen culture result identified *Pseudomonas aeruginosa*. The preoperative keratogram indicated that the preoperative corneal ulcer was located in the center of the pupil (A1). The corneal graft was highly edematous on day 1 after the surgery (A2). One month after the surgery, the corneal graft was almost transparent (A3). A4 shows the clear central graft 3 months after the surgery, but wing-like pseudopterygium could be seen on the nasal side. A5 shows the transparent corneal graft 12 months after the surgery, without any new vessels over the cornea. At the end of the follow-up, no corneal neovascularization, recurrence of the primary infection, or elevated intraocular pressure was observed. The visual acuity gradually improved to 0.3. 
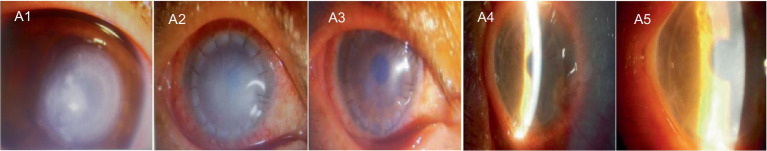


B1–B5 are images captured from another patient with a fungal corneal ulcer in the left eye. The pathogen culture result was positive for *Fusarium*. Image B1 shows the preoperative corneal ulcer located on the temporal side of the cornea. The corneal graft was highly edematous, and the sutures were moderately tight on day 1 after the surgery (B2). One month postoperatively, the corneal graft was almost transparent (B3). The graft center was transparent at 3 months postoperatively (B4). At 12 months postoperatively, the graft was completely transparent (B5). At the end of the follow-up, no neovascularization, recurrence of the primary infection, or elevated intraocular pressure was recorded. The visual acuity also gradually improved to 0.3. 
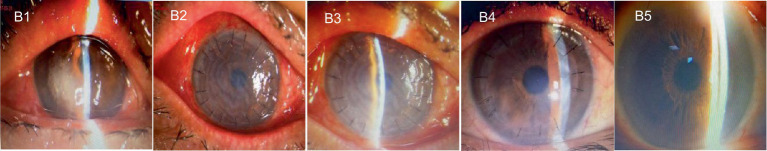


C1–C8 are images captured from a patient with a fungal corneal ulcer in the left eye. The pathogen culture result was positive for *Alternaria*. Image C1 shows the preoperative corneal ulcer located in the central cornea. Three months after the surgery, corneal graft rejection occurred (C2). After anti-rejection therapy, the corneal graft remained cloudy and the visual acuity did not improve 6 months after the surgery (C3). Then, another corneal transplantation was performed using a human donor cornea. Image C4 shows the transparent corneal graft 1 year after the second corneal transplantation. Image C5 shows the clear cornea with a cataract, 5 years after the surgery. The central corneal thickness was 463 um, and the morphology of the corneal endothelium was normal (C6 and C7). Image C8 shows the transparent corneal graft on day 1 after cataract surgery. During the entire follow-up period after the second corneal transplantation, no neovascular growth, recurrence of the primary lesion, or elevated intraocular pressure was recorded. The visual acuity increased from 0.08 to 0.4. 
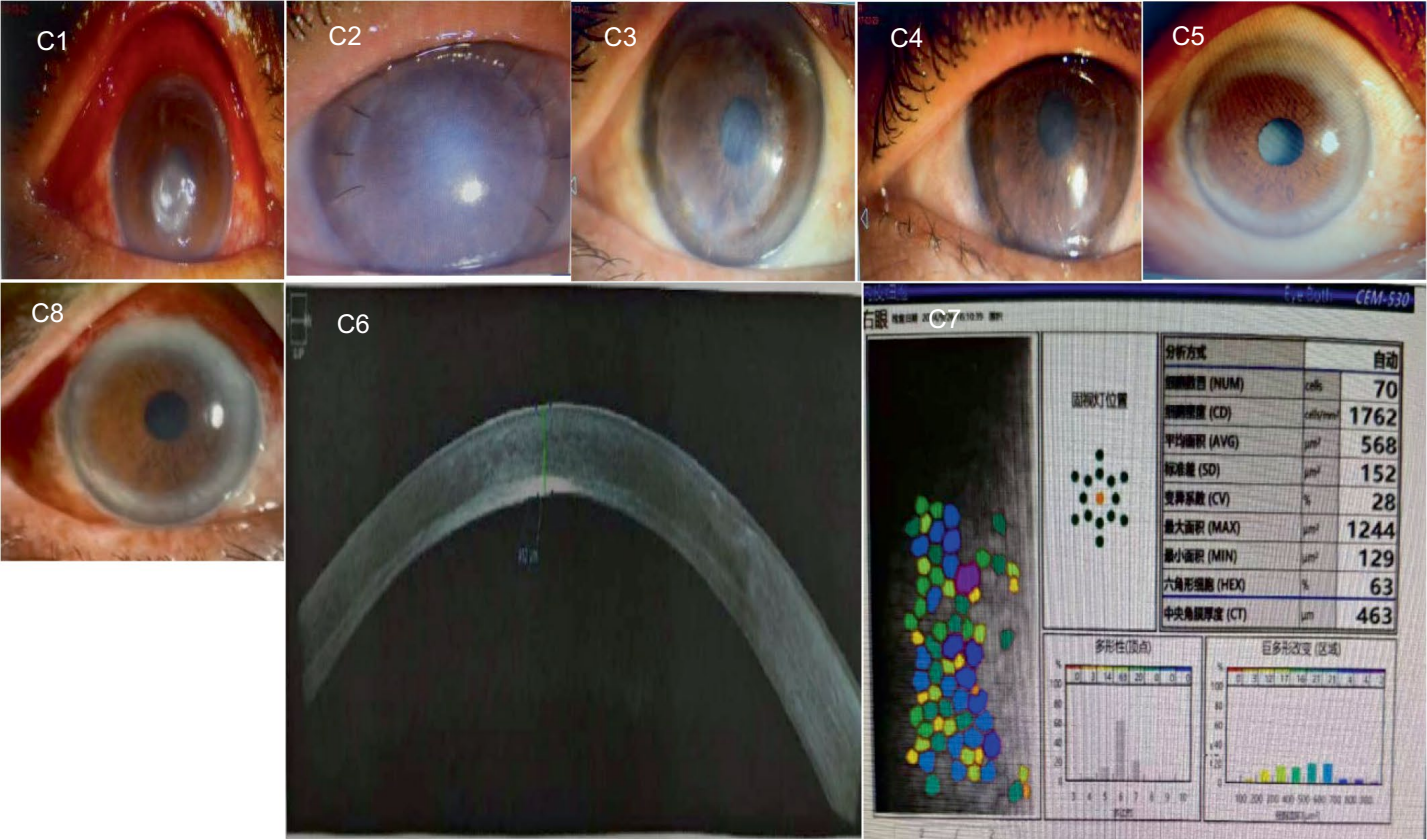


## 4 Discussion

The cornea is a transparent tissue in the eye’s refractive medium. When a lesion occurs, it can lead to a loss of transparency and may even spread to the eye, causing endophthalmitis and, eventually, the loss of the eyeball. Corneal blindness is one of the major causes of blindness in China, and corneal transplantation is an important surgery to restore vision (21). It is generally believed that corneal transplantation is more appropriate during the quiescent phase of the infection. However, in some patients who are in the active phase of the infection and have not responded to a period of anti-infective treatment, corneal transplantation may also be considered to remove the infected tissue. Some studies have reported that lamellar corneal transplantation is also safe and effective for treating infective keratitis, including virus keratitis and fungal keratitis (22, 23). However, the challenges of corneal transplantation today include the limited availability of corneal donors, transplant rejection, and recurrence of the original disease (24). In this study, we focused on the efficacy and safety of bioengineered corneas after implantation in humans. In this study, we observed severe edema in the early postoperative period in patients who received bioengineered corneas, On the one hand, tissue edema was due to tissue rehydration after the bioengineered corneal transplantation, and on the other hand, it was caused by postoperative inflammation. Corneal edema gradually resolves over the next 3 months. This is consistent with the recovery of APCS and the use of anti-inflammatory drugs, which may reflect the matrix remodeling process, corresponding to the change in transparency. The acellular porcine stroma graft may take at least 1 month or longer to restore transparency after corneal surgery. However, the graft remains less transparent compared to human donor corneas. The main reason for this is the disordered collagen fiber structure and the reduction in the refractive index of the interstitial medium. In addition, the decellularization process of bioengineered corneas may damage the collagen fibers (25), resulting in a disordered arrangement that can lead to fibrous scarring and ultimately corneal opacity (26, 1). It has been shown that donor corneal cells are gradually replaced by recipient corneal stromal cells after transplantation (27), and corneal stromal cells play a key role in corneal stromal remodeling (26), which can eventually transform the initially randomly oriented collagen fibers into highly ordered, staggered fibrous layers (28, 29). Therefore, the transparency of the graft slowly improves in the later stage, likely due to the activation of host stromal cells, which begin to deposit the extracellular matrix (ECM) and achieve a balance between collagen degradation and synthesis. In this study, corneal transplantation was performed on a patient with a fungal ulcer due to aggravated inflammation. The results showed that the corneal epithelium of this patient took a long time to fully heal and that the corneal stroma took a long time to restore transparency after the surgery. During the early postoperative period, the sutures became loosened, leading to graft rejection. Fortunately, the graft gradually restored its transparency after the administration of anti-inflammatory and immunosuppressive drugs. Another patient with a viral corneal ulcer did not follow the prescribed postoperative regimen and discontinued both topical and oral treatments. As a result, the ulcer recurred when he visited the hospital with complaints of foreign body sensation in his eye. Inflammation increases the release of collagenases, such as matrix metalloproteinases (MMPs), which accelerate collagen degradation and disrupt the reconstruction of the stroma (30). Therefore, preoperative and postoperative anti-inflammatory therapy and regular follow-up are crucial to reduce corneal ulcer recurrence and improve graft survival. Lifestyle factors, such as alcohol consumption and inadequate sleep, may contribute to persistent immune responses to residual viral antigens or virus-altered cellular proteins. Oral antiviral agents have been shown to reduce the recurrence rate of herpes simplex keratitis within 12 months, prolong the interval between recurrences, and shorten the duration of herpes outbreaks (31). It is also important to control the infection for more than 3 months before surgery (32) to ensure that the virus is in a quiescent state. It has been suggested that the corneal epithelium in the transplanted area can recover within 3 days, which is similar to the recovery observed with human corneal donors and consistent with the data from this study. However, there is debate over whether the diameter of the corneal graft using decellularized corneas should be 0.5 mm or 0.25 mm larger than the recipient stromal bed. In the first case of corneal transplantation using decellularized corneas, it was observed that as the edema of the graft subsided after surgery, the edge graft appeared slightly uneven and a pterygium invaded the cornea. This differed from the outcomes observed after conventional corneal transplantation, leading to an increase in inflammatory factors and the development of pseudopterygium. In most cases, the timing of suture removal is accelerated due to the loosening of sutures or neovascularization, which may be caused by the significant reduction in edema in postoperative corneal grafts, as well as the effects of decellularization reagents. These reagents may weaken collagen fibers, disrupt the microstructure of the extracellular matrix, and deteriorate its biomechanical stability (14), making the sutures more prone to loosing. If loose sutures are found during follow-up, they should be removed promptly to minimize the risk of infection and rejection, and contact lenses or corticosteroids should be used if necessary.

There are still many limitations to this study. Postoperative corneal nerve data were unavailable due to the lack of equipment. In addition, the sample size was too small and the follow-up period was not long enough to draw definitive conclusions.

In summary, our study supports the concept that bioengineered corneas, derived from acellular porcine corneas, are safe and effective alternatives to human cornea donors for the treatment of infectious corneal ulcers. However, future studies with a larger sample size and longer follow-up are needed to provide more robust and reliable clinical evidence. Bioengineered corneas offer a promising solution for corneal transplantation in cases of infectious corneal ulcers and can partially address the issue of cornea donor shortage.

## Data Availability

The raw data supporting the conclusions of this article will be made available by the authors, without undue reservation.
